# Children’s Attitudes Toward Dementia: Protocol for a Cross-Sectional Study

**DOI:** 10.2196/81576

**Published:** 2026-04-20

**Authors:** Gema Somoza-Fernández, Javier Ortiz-Alonso, José Antonio Serra-Rexach, María Teresa Vidán

**Affiliations:** 1 Geriatrics Department, Hospital General Universitario Gregorio Marañón, Calle Doctor Esquerdo 46, Madrid, 28007, Spain, 34 915866704; 2 Biopathology of Aging Group, Instituto de Investigacion Sanitaria Hospital General Universitario Gregorio Marañón, Madrid, Spain; 3 Biomedical Research Networking Center on Frailty and Healthy Aging (CIBERFES), Instituto de Salud Carlos III, Madrid, Spain; 4 Faculty of Medicine, Universidad Complutense de Madrid, Madrid, Spain

**Keywords:** attitudes, knowledge, dementia, children, surveys and questionnaires

## Abstract

**Background:**

Research on dementia has primarily focused on adults, leaving a gap in understanding related to children’s attitudes toward dementia. This gap is relevant given the growing societal impact of dementia and the potential role of early education in shaping attitudes.

**Objective:**

The primary objective of this study is to assess attitudes toward dementia in a sample of Spanish primary school children. Secondary objectives include (1) examining the psychometric properties of the questionnaire developed for this study, (2) identifying factors associated with differing attitudes toward dementia, and (3) evaluating children’s social desirability and its correlation with questionnaire responses.

**Methods:**

A 23-item, tripartite Likert-type questionnaire (Cuestionario de Actitudes de los Niños Españoles Hacia la Demencia, or “Spanish Children’s Attitudes Toward Dementia Questionnaire”) was developed through a comprehensive literature review, a pilot study with a convenience sample of 10 children, and expert panel consultation. The study sample will comprise children aged 8 to 11 years enrolled in the fourth and fifth grades at 6 selected schools in the Community of Madrid, Spain. Participants will complete the questionnaire alongside a social desirability scale. Additional variables (eg, current or previous cohabitation with grandparents) will be collected to explore factors associated with differing attitudes toward dementia.

**Results:**

This study is not funded. Data collection began in January 2025 and is projected to conclude in January 2026. As of July 2025, a total of 164 participants from 3 selected schools have been recruited. Data analysis is expected to begin in January 2026, and results are planned for publication in 2027.

**Conclusions:**

This study represents a feasible and novel initiative that may enhance understanding of children’s attitudes toward dementia. The findings could inform the development of targeted educational interventions and support the inclusion of dementia-related content in school-based education.

## Introduction

### Background

Dementia is a leading cause of disability among older individuals worldwide [1]. Current estimates suggest that approximately 55 million individuals are living with dementia worldwide, a figure projected to increase to 150.8 million by 2050 [2]. Dementia affects not only the quality of life of those diagnosed but also that of their caregivers and families, as caregiving often entails personal, psychological, and financial challenges [3].

To address this global health issue, the *Global Action Plan on the Public Health Response to Dementia 2017-2025* was unanimously adopted by all 194 member states of the World Health Organization at the 70th World Health Assembly. The objective of this initiative is to foster a society in which individuals with dementia and their caregivers can realize their full potential with dignity, respect, and equality [4].

Despite these objectives, significant gaps remain in knowledge and awareness of dementia. This is evident in the widespread misconception of the disease as either reversible [5] or an unavoidable consequence of aging that cannot be prevented [6]. Furthermore, it is widely recognized that the general public often experiences discomfort and shame in the presence of individuals with dementia, contributing to the stigma surrounding the condition [7]. For instance, a 2012 survey involving 127 individuals with dementia and 1716 caregivers revealed that 25% and 11% of respondents, respectively, had concealed the diagnosis from friends or family members due to stigma [8].

While research on dementia perception has primarily focused on adults, there remains a significant gap in understanding how children perceive this condition. This gap is critical given that children represent the future of a society increasingly affected by the growing prevalence of dementia and related challenges. Considering that childhood is a critical period for instilling knowledge and values [9], addressing this gap is imperative.

Research on children’s feelings and behaviors regarding dementia has mainly focused on the impact of the disease on grandparent-grandchild relationships [10-12]. Regarding knowledge about the disease, most children and adolescents obtain information about dementia from media sources [13], as parents often avoid discussing dementia-related topics with their children [14]. However, several educational interventions over the past decade have successfully improved children’s understanding and knowledge of dementia [15-18]. Similarly, positive outcomes have been reported from “intergenerational interventions” [19-21], which involve direct interactions between children and individuals living with dementia.

### Study Hypothesis

The central hypothesis of this study is that children’s feelings and behaviors regarding dementia are largely unknown and that their level of disease-related knowledge is generally low. These 3 aspects—affective, behavioral, and cognitive responses toward a person, situation, or object—constitute the widely accepted tripartite model of “attitude” [22]. Accordingly, this study aims to examine children’s attitudes toward dementia, as well as the 3 domains of this construct, in a targeted sample of primary school children.

### Study Objectives

The primary objective of this study is to assess children’s overall attitude toward dementia and its 3 domains—feelings, behaviors, and knowledge about the disease—among fourth- and fifth-grade primary school students from 6 selected schools in the Community of Madrid, Spain.

Secondary objectives include the following:

- Examining the psychometric properties of the questionnaire developed for the main objective
- Identifying factors associated with differing dementia-related attitudes, both overall and across the 3 domains (feelings, behaviors, and knowledge about the disease)
- Determining children’s social desirability and its correlation with the questionnaire responses

## Methods

### Study Design

This is a quantitative, cross-sectional study that will be conducted between January 2025 and January 2026.

### Study Participants and Setting

As mentioned previously, the study sample will consist of children enrolled in fourth and fifth grade of primary education (aged 8-11 years) in 6 selected schools in the Community of Madrid.

Schools will be purposively selected to reflect diversity in socioeconomic context, educational management, and religious orientation. As a first step, regions in the Community of Madrid will be ranked according to area-level socioeconomic status [23] and divided into 2 strata representing the lower and upper halves of the distribution. Subsequently, within each stratum, 1 public school, 1 secular private or concerted school, and 1 private or concerted school with religious education will be selected.

Schools will be contacted via email and provided with an information sheet describing the study aims and procedures; those that agree to participate will then be scheduled for data collection visits. If a school declines participation, a replacement school from the same stratum with the same educational management and religious orientation will be invited.

### Sample Size

As will be described below, the main primary outcome of this study is the total score on a 23-item, Likert-type questionnaire (5 response options per item; maximum total score=115) measuring attitude toward dementia. Therefore, the sample size was calculated using the Cochran formula for estimating a mean (continuous outcome) [24,25]:


$$n_{o}=\frac{Z^{2}\cdot SD^{2}}{d^{2}}$$

In this formula, *Z* is 1.96 (95% confidence), *SD* is 26 (a conservative estimate of the SD of the total questionnaire score), and *d* is 3.45 points (3% of the maximum score of 115). This yields 219 evaluable children (n₀).

Finally, an explicit adjustment for nonevaluable questionnaires was incorporated. We anticipated 15% to 20% nonevaluable questionnaires (eg, parental consent not returned or declined, absence on the survey day, or child nonassent) and inflated the sample size using the standard approach [26]:


$$N=\frac{n_{o}}{\left(1-r\right)}$$

In this formula, *N* is the target number to recruit, *n_₀_* is the minimum required number of evaluable participants, and *r* is the anticipated proportion of nonevaluable cases (0.15-0.20). This yields 260 to 270 children.

### Inclusion Criteria

All children currently attending fourth or fifth grade of primary education in the selected schools will be eligible to participate in the study provided they meet the following criteria: (1) provision of written consent from their parents or legal guardians (opt-in procedure) in accordance with Spanish Law 26/2015 of July 28 on the modification of the system of protection for childhood and adolescence and (2) provision of verbal assent immediately prior to participation and only if parental or guardian consent has been obtained.

### Primary Outcomes

#### Overview

Given the absence of a validated scale in Spain to assess attitudes toward dementia in children, a questionnaire was developed. As described above, the construct of “attitude” comprises an individual’s cognitive, behavioral, and affective response to an object, person, or situation. This tripartite model served as the foundation for the questionnaire developed for this study, named the Cuestionario de Actitudes de los Niños Españoles Hacia la Demencia (CANDem; “Spanish Children’s Attitudes Toward Dementia Questionnaire”).

The CANDem comprises 23 items on a 5-point Likert scale related to the 3 domains of the tripartite model: 12 cognitive items (eg, “All people develop dementia as they age”), 6 affective items (eg, “It would be a very boring experience to spend an entire afternoon with a person with dementia”), and 5 behavioral items (eg, “If a person with dementia forgot something important, I would get a little angry so that they remember it better next time”). The maximum total score is 115, with higher scores indicating more positive attitudes toward dementia.

The main primary outcome will be the total score on the CANDem, which reflects overall attitudes toward dementia. The separate scores on each domain (cognitive, affective, and behavioral) will be considered additional primary outcomes.

The CANDem was developed through the following stages: (1) content development, (2) measure design, (3) expert panel review, (4) pilot-testing, and (5) re-evaluation by the expert panel.

#### Phase 1: Content Development

This phase involved identifying relevant domains for the questionnaire, informed by a comprehensive review of prior studies, expert opinions, and methodological guidelines for attitude questionnaires [27].

##### Previous Studies

Item selection was based on previously validated dementia attitude scales:

- Dementia Attitudes Scale—a scale comprising 20 items on a 7-point Likert scale measuring attitudes toward Alzheimer disease among American college students and care workers, demonstrating strong reliability and a 2-factor structure (“dementia knowledge” and “social comfort”) [28]
- Escala de Actitudes Hacia la Demencia (“Attitudes Toward Dementia Scale”)—a scale comprising 20 items on a 7-point Likert scale validated among Argentine psychology, medical, and nursing students showing good reliability and a 3-factor structure (“rejection,” “affection,” and “experience”) [29]
- Kids Insight Into Dementia Survey—a scale comprising 14 items on a 5-point Likert scale developed in Australia to assess children’s perceptions of dementia, with good reliability and a 3-factor structure (“personhood,” “stigma,” and “dementia understanding”) [30]
- Dementia Community Attitudes Questionnaire—a 10-item Likert-type scale assessing attitudes toward dementia in the general population comprising 3 factors (“engagement,” “challenges,” and “decision-making”) [31]

Additionally, findings from a qualitative study were considered [32]. In this study, 27 participants with personal experience of dementia—including 12 primary caregivers, 4 nonprimary caregivers, 6 grandchildren of people with dementia, and 5 individuals with early-stage dementia—were asked what they considered most important for children to learn about the disease.

##### Item Formulation

Items were designed to be brief, clear, concise, and relevant.

##### Number of Items

An initial pool of items exceeding the expected final number was generated to allow for item reduction during subsequent stages.

In consideration of the aforementioned recommendations, a preliminary 24-item questionnaire was constructed. Items addressed themes such as the dignity of individuals with dementia (eg, that they can still enjoy life and feel the love of their family and friends); the importance of nonpharmacological interventions; lifestyle habits potentially influencing dementia risk; and common misconceptions, including the beliefs that dementia is inevitable, contagious, or reversible.

### Phase 2: Measure Design

Given the preference for Likert scales over dichotomous items in attitude measurement [33], a 24-item questionnaire with a Likert-type response format was selected. Regarding the number of response categories, there is evidence suggesting that scales comprising 2-, 3-, and 4-point options tend to perform suboptimally compared to those with more response options [34]. Therefore, each item was rated using a 5-point Likert-type scale including an “I don’t know” option. Affective and behavioral items were rated as follows: “strongly agree” (5), “agree” (4), “I don’t know” (3), “disagree” (2), and “strongly disagree” (1). For cognitive (knowledge) items, the same 5-point response options were used; however, “I don’t know” responses will be treated as missing rather than as the midpoint as they indicate absence of knowledge rather than a neutral opinion. The proportion of “I don’t know” responses will be reported given that they provide relevant information about children’s understanding.

The inclusion of the “I don’t know” option aimed to reduce arbitrary guessing and social desirability bias [35]. Additionally, to mitigate acquiescence bias, which refers to the tendency to select positive responses, 13 items are reverse scored [36].

### Phase 3: Review by a Panel of Experts

A panel of 7 dementia experts evaluated the 24 questionnaire items for clarity and content validity using a structured evaluation framework [37]. The panel included 6 geriatricians with academic, clinical, and research expertise, as well as 1 individual with lived experience as a long-term caregiver for a family member with Alzheimer disease. In addition, to ensure that the questionnaire was age appropriate, 3 primary school teachers and 2 pediatricians contributed to the adaptation of item wording into child-friendly language.

The panel reached a consensus on the final set of 24 items. Personal data were protected in accordance with Spain’s Organic Law 3/2018 on the Protection of Personal Data and Guarantee of Digital Rights.

### Phase 4: Pilot-Testing

The aim of this phase was not to evaluate participants’ responses but rather to identify potential issues related to item wording and comprehension. Accordingly, a relatively small sample size was considered appropriate in line with previous methodological recommendations [27]. A convenience sample of 10 children aged 8 to 11 years was recruited. Prior to participation, parents or legal guardians were provided with an information sheet and asked to sign a consent form.

The questionnaire was administered in paper format by one of the study authors at the participants’ homes. Each child provided verbal assent immediately before starting the questionnaire in accordance with ethical procedures. The questionnaire was completed in a quiet setting in a one-to-one manner with the researcher to ensure privacy and minimize external influence. Parents or guardians were asked not to be present during completion. The researcher was available throughout to clarify any questions or explain unfamiliar terms.

Before starting, children were asked whether they were familiar with the terms “dementia” or “Alzheimer’s.” If not, the researcher provided a brief, neutral explanation to support basic understanding. Care was taken to avoid influencing responses to the questionnaire items.

Children described the questionnaire as “simple” and “easy to understand.” Comprehension difficulties were noted in items 10, 18, and 23. The estimated completion time was 20 minutes. After finishing, the children expressed a desire to learn more about dementia and asked questions about the disease, including the possibility of a cure, the impact of dementia on an individual’s ability to bathe or dress, and the most effective treatment for an anxious person with dementia.

### Phase 5: Re-Evaluation by the Panel of Experts

On the basis of the results of the pilot study, this phase aimed to finalize the CANDem. The expert panel reviewed the pilot findings and made the necessary modifications. In accordance with previous studies [30], items with more than 50% of extreme responses (ie, “strongly agree” or “strongly disagree”) were excluded to avoid floor or ceiling effects.

Items 10, 18, and 23 were revised to enhance clarity and comprehension. Item 24 (“I find it is sadder to have dementia than to fall and break an arm”) was removed as 80% of the children selected the “strongly agree” option.

Given that 4 out of 10 children in the pilot study demonstrated difficulty understanding the term “dementia,” the expert panel decided to introduce the final version of the CANDem with selected vignettes from the Spanish children’s comic “You, Me, and Alzheimer’s Disease,” which depicts a person experiencing temporal disorientation and short-term memory loss [38]. From the comic, vignettes were chosen that did not provide clues or otherwise influence children’s responses to the questionnaire, thereby minimizing potential framing effects.

Textbox 1 presents the final version of the CANDem. To facilitate comprehension of this manuscript, the questionnaire has been translated into English from its original Spanish format. For this purpose, a formal translation and back translation procedure was applied. First, a bilingual researcher translated the Spanish version into English. Second, a graduate in translation and interpreting independently back translated the English version into Spanish. Finally, the research team compared the back translation with the original Spanish version and reconciled any discrepancies to ensure equivalence of meaning.

Textbox 1. **Final version of the CANDem (Cuestionario de Actitudes de los Niños Españoles Hacia la Demencia*,* or “Spanish Children’s Attitudes Toward Dementia Questionnaire”).**

- Item 1: “All people develop dementia as they age” (cognitive item; reverse scored)
- Item 2: “It would be a very boring experience to spend an entire afternoon with a person with dementia” (affective item; reverse scored)
- Item 3: “There are no medications that can cure dementia” (cognitive item)
- Item 4: “If I were introduced to a person with dementia, I would try to spend time with them to get to know them better” (behavioral item)
- Item 5: “I would not feel upset if a family member or friend with dementia forgot my birthday” (affective item)
- Item 6: “A person with dementia may transmit it to another person” (cognitive item; reverse scored)
- Item 7: “It is possible to adopt certain habits (such as not smoking or exercising) that can help to prevent dementia” (cognitive item)
- Item 8: “If I met someone with dementia, I would speak to them as if they were little children to facilitate their understanding” (behavioral item; reverse scored)
- Item 9: “People with dementia are sad almost all of the time” (cognitive item; reverse scored)
- Item 10: “I would not be scared if I saw someone with dementia being nervous and shouting” (affective item)
- Item 11: “If a person with dementia forgot something important, I would get a little angry so that they remember it better next time” (behavioral item; reverse scored)
- Item 12: “Dementia can impair a person’s ability to perform activities such as showering, dressing, or walking” (cognitive item)
- Item 13: “If someone with dementia kept asking me the same question all the time (for example, ‘What day of the week is it today?’), I would start laughing” (behavioral item; reverse scored)
- Item 14: “People with dementia often do not perceive displays of affection from their family or friends” (cognitive item; reverse scored)
- Item 15: “If I were walking down the street in a hurry and saw someone with dementia who was lost, I would stop to help them” (behavioral item)
- Item 16: “People with dementia often have poor hearing, and that’s why it’s very important to speak to them loudly” (cognitive item; reverse scored)
- Item 17: “If someone with dementia gets angry or nervous, we must give them a pill to calm them down and then talk to them” (cognitive item; reverse scored)
- Item 18: “Puzzles and music are activities that can help people with dementia” (cognitive item)
- Item 19: “People with dementia can recover from the disease and go back to being themselves” (cognitive item; reverse scored)
- Item 20: “If I had a family member with dementia, I would not be embarrassed to tell my friends” (affective item)
- Item 21: “I wouldn’t like to play a game with someone with dementia because they would forget the rules all the time” (affective item; reverse scored)
- Item 22: “I would get a little nervous if a person with dementia smiled at me” (affective item; reverse scored)
- Item 23: “People with dementia can have hobbies or enjoy their free time” (cognitive item)

### Secondary Outcomes

Secondary outcomes will provide additional information about children’s attitudes toward dementia and the psychometric performance of the CANDem. The first secondary outcome is psychometric properties of the CANDem, including internal consistency, item discrimination, and construct and content validity. Additional details on the statistical evaluation of these properties are provided in the Statistical Analysis section. The second outcome is children’s social desirability, defined as the tendency to respond in culturally appropriate or socially approved ways. This will be measured using an 8-item dichotomous (“yes” or “no”) scale previously validated for use in Spanish-speaking children, with higher scores indicating greater social desirability [39]. This scale will be administered together with the CANDem in paper format. Details on how associations between social desirability and CANDem scores will be examined are provided in the Statistical Analysis section. The third outcome is factors associated with attitudes toward dementia. The following variables will be collected: age, sex, country and city of birth, parents’ country of birth, prior familiarity with the term “dementia” (ie, whether the child had heard of the term before the survey session), prior knowledge of its meaning (ie, whether the child knew the meaning of the term before the survey session), presence of a family member or acquaintance with dementia, previous cohabitation with a person with dementia, and previous cohabitation with grandparents or other older adults. These variables will be completed by the child after finishing both the CANDem and the social desirability scale, and their relationships with differing dementia-related attitudes—both overall and across the 3 domains (feelings, behaviors, and knowledge about the disease)—will be assessed using regression models (see the Statistical Analysis section).

### Ethical Considerations

This study was reviewed and approved by the ethics committee of Gregorio Marañón University Hospital (approval Act 18/2023; October 9, 2023).

A few days before the researcher visits a school to administer the questionnaire, the parents or legal guardians of all fourth- and fifth-grade students at the corresponding school will receive a participant information sheet and the informed consent form for study participation. On the day of the survey, immediately before questionnaire administration, children whose parents or legal guardians have given written consent will receive age-appropriate information and be asked to provide verbal assent. Only children with both written parental or legal guardian consent and their own verbal assent will participate in the study following an opt-in procedure. No compensation will be provided to participants.

All data will be collected, processed, and stored in accordance with the General Data Protection Regulation. Study data will be anonymized prior to analysis to ensure participant confidentiality. The study will be conducted in accordance with the principles of the Declaration of Helsinki and will follow JMIR [40] and Committee on Publication Ethics (COPE) ethical guidelines [41].

### Data Collection Procedure

Data collection will take place during prescheduled visits coordinated with each school to minimize disruption to normal academic activities. Only children for whom written parental or legal guardian consent has been obtained and who provide verbal assent immediately prior to participation will receive an individual paper-based booklet containing (1) the vignettes presenting a person with dementia, (2) the CANDem, (3) the social desirability scale, and (4) the section covering sociodemographic and dementia-related exposure variables. At the end of the booklet, children will respond to 2 brief acceptability items—“Was the questionnaire easy to complete?” and “Would you like to learn more about dementia?”—both with dichotomous (“yes” or “no”) response options.

Before beginning the survey, children will be reminded that their participation is anonymous, that they should answer honestly, and that they may use the “I don’t know” option whenever they are unsure. The classroom teacher will remain present throughout the session, and the researcher will be available to clarify procedural questions without influencing responses.

As demonstrated in the pilot study, completing the CANDem will take approximately 20 minutes. As the additional components of the booklet—reading the vignettes, completing the social desirability scale, and responding to the sociodemographic and dementia-related exposure questions—were not timed during the pilot study, each classroom session is planned to last approximately 1 hour. This includes distribution of materials, completion of all components, and a brief 10-minute discussion at the end during which children may ask questions and provide feedback on any difficulties encountered with the questionnaire.

A child distress protocol will be implemented to safeguard participants’ well-being. Should any child show signs of discomfort (eg, if the content evokes personal experiences, such as having a family member with dementia), the researcher will provide supportive listening, immediately inform the classroom teacher, and pause the questionnaire for that child.

### Statistical Analysis

All statistical analyses will be conducted using SPSS Statistics (version 25; IBM Corp) or other equivalent statistical software. A 2-sided significance level at a *P* value of .05 will be applied for prespecified analyses.

#### Clustering and School Effects

Because children are nested within classrooms and schools, regression-based inferential analyses will account for clustering. Given that only 6 schools are included, multilevel models with random effects cannot be reliably estimated [42,43]; therefore, school will be included as a fixed-effect variable, and within-classroom dependency will be addressed using robust SEs clustered at the classroom level. For planning purposes, an intraclass correlation coefficient of 0.05 will be assumed, consistent with typical values in educational research [44].

#### Primary Outcomes

As noted, the primary outcomes of this study are the CANDem total score and the scores on its 3 domains (cognitive, affective, and behavioral). Descriptive statistics, including means, SDs, medians, IQRs, and observed ranges, will be calculated for the total score and each domain.

#### Secondary Outcomes

As stated above, secondary outcomes include the psychometric properties of the CANDem, children’s social desirability, and factors associated with differing attitudes toward dementia.

##### Psychometric Properties of the CANDem

Internal consistency will be evaluated using the Cronbach α for the total score and each domain, with values above 0.70 considered acceptable [36,45]. Item discrimination will be assessed using the corrected homogeneity index, calculated as the Pearson correlation between each item and (1) its corresponding domain score and (2) the total score, excluding the item under evaluation; items with a homogeneity index of 0.20 or lower will be considered to show poor discrimination [46]. Construct validity will be examined through exploratory factor analysis (EFA) using principal axis factoring after verifying sampling adequacy. Content validity has already been ensured through a rigorous development process, including an exhaustive literature review, consultation with an expert panel, and pilot-testing.

As the CANDem has been translated into English to facilitate understanding of this manuscript, it is important to note that all psychometric analyses will be conducted exclusively on the Spanish version of the instrument, which is the language in which it will be administered to participants.

##### Children’s Social Desirability

Scores on the 8-item dichotomous scale will be analyzed. Associations between social desirability and CANDem scores will be described using Pearson correlation coefficients. For inferential purposes, associations will also be examined using linear regression models, with school included as a fixed effect and robust SEs clustered at the classroom level.

##### Factors Associated With Children’s Attitudes Toward Dementia

The sociodemographic and contextual variables described in the Secondary Outcomes section (eg, age, sex, previous cohabitation with grandparents or other older adults, and prior exposure to dementia) will first be described using appropriate descriptive statistics.

Subsequently, to explore whether these variables are associated with differing attitudes toward dementia, the CANDem total score and its 3 domain scores (cognitive, affective, and behavioral) will be analyzed as continuous outcomes using linear regression models accounting for school effects and clustering, as described above. In addition, the total score and domain scores will be categorized into quartiles, and associations with the same predictors will be examined using ordinal logistic regression models (proportional odds), likewise accounting for school effects and clustering. The proportional odds assumption will be assessed.

Linearity (where applicable), homoscedasticity, and collinearity will be assessed for all regression models, and appropriate adjustments will be applied if necessary.

### Exploratory Analyses

Exploratory analyses will compare the CANDem total score and domain scores across prespecified subgroups (age group, sex, child’s country and city of birth, parents’ country of birth, prior familiarity with the term “dementia,” prior knowledge of its meaning, presence of a family member or acquaintance with dementia, previous cohabitation with a person with dementia, previous cohabitation with grandparents or other older adults, and school type), with corresponding *P* values reported.

In addition, item-level response patterns will be described by reporting the percentage distribution across the 5 Likert response options for each CANDem item. Item-level responses will be treated as ordinal variables, and differences across subgroups will be explored by comparing response distributions. All exploratory analyses will be clearly labeled as such. When multiple hypothesis tests are performed within exploratory analyses, *P* values will be adjusted to control the false discovery rate at 0.05.

### Missing Data

The extent of missing data will be reported for all study variables. As described above, “I don’t know” responses to cognitive (knowledge) items will be treated as missing, and their frequency will be reported.

Descriptive analyses will generally use available-case data for each variable so that participants with missing responses on a given measure can still contribute to analyses of other variables. For regression-based analyses, complete-case analyses will be performed for the variables included in each model. Missingness below 5% will be considered negligible. If more than 10% of values are missing for any key variable, patterns of missingness will be examined, and results will be interpreted cautiously.

## Results

This study is not funded. Data collection began in January 2025 and is projected to conclude in January 2026. As of July 2025, a total of 164 participants from 3 selected schools have been recruited. The study has been well received, as reflected by the fact that all children with parental consent have provided verbal assent and participated. There has been no need to activate the child distress protocol. Data analysis is expected to begin in January 2026, and results are planned for publication in 2027.

## Discussion

### Study Rationale and Limitations

This study is among the first in Spain to evaluate attitudes toward dementia among schoolchildren. The proposed design and the pilot phase demonstrate the feasibility of the project.

Investigating this issue in a country such as Spain—one of the nations with the highest aging rates worldwide—is critically important. Over the past 5 decades, societal changes have led to a decline in intergenerational cohabitation, with most older adults no longer living with younger family members. As a result, children today are increasingly unfamiliar with aging and age-related diseases such as dementia.

As previously noted, due to the lack of a validated Spanish-language scale to assess children’s attitudes toward dementia, a new questionnaire was developed. One prior study in Spain explored the emotional experiences and relational changes between grandchildren and grandparents with dementia, resulting in the development of a specific tool for that context [10]. However, its focus differed significantly as it addressed personal emotions such as guilt, jealousy, and satisfaction derived from helping without examining other domains of attitudes. Additionally, that study targeted adolescents and young adults aged 14 to 21 years, making it not directly comparable to this study.

The CANDem comprises cognitive, affective, and behavioral items. Compared with other dementia attitude scales validated in children and adolescents [15,47,48], this questionnaire includes a greater number of items assessing children’s knowledge about the disease (eg, “All people develop dementia as they get older” or “There are no drugs that can cure dementia”). The decision to prioritize the assessment of knowledge over the other domains of attitude was informed by the well-established link between understanding dementia and subsequent feelings and behaviors toward people living with the condition [7]. Furthermore, knowledge is arguably the most modifiable component through school-based interventions and plays a critical role in shaping children’s values and attitudes.

Participants in the pilot study completed the questionnaire within 20 minutes and described it as clear and easy to understand, supporting its feasibility for use with the selected age group. The children’s strong interest in learning more about dementia aligns with findings from a British survey in which 62% of children expressed a desire to help people with dementia but reported that a lack of understanding limited their ability to do so. Additionally, more than half of the surveyed children believed that life for people with dementia would improve if the general public had more knowledge about the condition [49].

Although the psychometric evaluation of the questionnaire is not the primary focus of this study, we considered it important to assess its internal consistency and validity.

Several limitations should be acknowledged; one of the most important ones is that some items in the CANDem may reflect common stereotypes about individuals with dementia (eg, “If someone with dementia gets angry or nervous, we must give them a pill to calm them down” or “People with dementia are sad almost all of the time”). These items were included to capture children’s real attitudes, including misconceptions, rather than to reinforce or endorse such beliefs. To minimize potential bias, all items are presented neutrally, and care was taken during pilot-testing to ensure that they were age appropriate and clearly understood. Furthermore, after completing the survey, a 10-minute discussion session will be held in each classroom to allow children to ask questions and reflect on the items. While these precautions have been implemented, the inclusion of potentially stereotype-related items may have influenced responses in some children and may affect the interpretation of the findings.

Methodological limitations should also be noted. First, the cross-sectional design precludes causal inference, so relationships between attitudes and other variables cannot be interpreted as causal. Second, social desirability bias may influence children’s responses. Despite efforts to present items neutrally and ensure a safe and age-appropriate survey environment, participants may answer in ways they perceive as socially acceptable rather than fully reflecting their true attitudes. To address this, the CANDem will be administered together with a child social desirability scale to assess the potential influence of this bias. Third, the psychometric evaluation of the CANDem will rely on EFA to identify its underlying structure. While EFA is appropriate for initial validation, future studies should conduct confirmatory factor analysis to test the stability and robustness of the factor structure derived from this work. Finally, the generalizability of the findings may be limited as the study is being conducted in schools within the Community of Madrid, and the CANDem may require adaptation for use in cultural contexts outside Spain considering differences in educational systems and family structures.

### Conclusions and Implications

The proposed study appears to be a viable project that will contribute to a deeper understanding of children’s attitudes toward dementia in Spain. By identifying existing attitudes and associated factors, it may inform the development of targeted interventions within the Spanish educational system aimed at increasing awareness and empathy among younger populations. These findings could also guide educational policy by supporting the inclusion of dementia-related content in school curricula, thereby fostering more inclusive and compassionate communities and potentially improving the care and quality of life of individuals living with dementia.

## Abbreviations

CANDem: Cuestionario de Actitudes de los Niños Españoles Hacia la Demencia (Spanish Children’s Attitudes Toward Dementia Questionnaire)
COPE: Committee on Publication Ethics
EFA: exploratory factor analysis
